# A meta-analysis on the effect of implant characteristics on the survival of the wide-diameter implant

**DOI:** 10.1186/s40729-015-0030-2

**Published:** 2015-11-03

**Authors:** Miriam Ting, Matthew Palermo, David P. Donatelli, John P. Gaughan, Jon B. Suzuki, Steven R. Jefferies

**Affiliations:** 1 Kornberg School of Dentistry, Temple University, 3223 North Broad Street, Philadelphia, PA 19140 USA; 2 School of Medicine, Temple University, 3420 N Broad St, Philadelphia, PA 19140 USA

**Keywords:** Wide-diameter implants, Surface-treated or machined implants, Short implants or long implants, Implants in the mandible or maxilla

## Abstract

The purposes of the study are to study the implant survival of the wide-diameter implant and to analyze if the length, the implant surface, or the placement location has any effect on its survival. Electronic databases were searched from inception to Dec 2014. Studies included in the review had implants placed in areas of adequate bone width and had clear inclusion and exclusion criteria for patient selection. Immediately placed and immediately loaded implants were excluded. A meta-analysis was done using the “random effects” model on the included studies. And, a meta-regression was used to evaluate the effects of location, length, and surface on the implant survival. Of the six studies selected, three evaluated surface-treated implants and three machined implants. The overall pooled survival rate of the wide implant is 96.3 %. The meta-regression showed that when using a wide implant, neither its surface nor its length nor its position in the maxilla or mandible adversely affected its survival (*P* > 0.05). This meta-analysis concluded that the location, length, and surface of the wide-diameter implant did not affect its survival and therefore suggested that when the conditions of the implant site corresponded to the inclusion criteria of our meta-analysis, choosing a wide-diameter implant in the posterior mandible or maxilla, where implant length may be limited by the nerve or the sinus, the use of a short implant regardless of its surface would not affect its survival.

## Review

### Introduction

Endosseous implants were used reliably in the treatment of various degrees of edentulism [1–7]. In restoring the edentulous ridge, the clinician could be faced with difficult bony situations. The wide-diameter implant could be used in these situations to improve primary stability by increasing the surface area available for osteointegration [8–10]. Biomechanically, the wide-diameter implant engaged maximal bone, increased initial stability, and improved stress distribution in the supporting bone [11]. In restoring a large molar, the wide implant has the added advantage of increasing the load bearing capacity and emergence profile of the final restoration [10]. It has been shown to be three to six times stronger than the standard implant [12]. Wide-diameter implants were also used as rescue fixtures to replace fractured or nonintegrated implants [8]. Thus, the wide-diameter implants could become the implant of choice when faced with these challenging situations.

The aims of this review were to research the literature published till Dec. 15, 2014, on wide-diameter implants and to perform a meta-analysis to study (1) the wide-diameter implant survival of different lengths, (2) the wide-diameter implant survival of modified surface compared to machined surface, and (3) the implant survival of wide-diameter implants placed in the maxilla compared to the mandible.

### Materials and methods

#### Focused question

1. Does length of the wide-diameter implant influence its survival?
2. Does the surface modification influence its survival compared to machined implant surfaces?
3. Does the implant placement in the maxilla or the mandible influence its survival?

#### Literature search and study design

The database on PubMed, Web of Science, and Cochrane Central Register of Controlled Trials was searched from inception to December 15, 2014. The keywords for the search were “dental implants or dental implant” and “wide,” and a reference librarian was consulted as to the most effective strategy. Gray literature was also searched on Google Scholar using advance search to find articles with all of the words “wide, dental, implants, endosseous, clinical, patients, survival” and without the words “animal, graft, augmentation, immediate, review”. Hand searching was conducted on the reference lists of identified wide-diameter implant articles and was limited to articles not already identified in the above search strategy. Implant representatives of implant manufacturers were also contacted for any ongoing research pertaining to wide-diameter implants, and researchers were invited to clarify research information.

#### Inclusion/exclusion criteria

1. Randomized controlled trials, controlled clinical trials, cohort, and case series reporting on the implant survival of wide-diameter endosseous titanium implants with different surface modifications were included. Only prospective data were included. Case reports, conventional reviews, and systemic reviews were excluded.
2. Implant diameter greater or equal to 4.7 mm were considered wide-diameter implants.
3. Only articles with specific documentation for wide-diameter implants were included. This documentation includes implant length, location/site, loading times, and specific failure data such as length, location, and timing of failure for wide-diameter implants.
4. Articles with information on implants placed in sites deemed to have adequate bone height and width, and did not require site development, were included. Articles with grafted sites and/or unclear description of how sites were selected were excluded.
5. Only articles with data on wide implants loaded after least 1–3 months of healing after implant placement were included; data on immediate placement in extraction sites and immediately loading implants were not covered in this review.
6. Wide-diameter implants used in immediate replacement of failed implants were excluded.
7. Studies with at least 1-year follow-up and included at least 10 implants regardless of diameter and length were included.
8. Patients with adequate health to undergo implant surgery and patients with controlled medical conditions were not excluded.
9. Smoking status of subjects was not considered a criterion for exclusion.
10. Non-English articles or articles without English translations were excluded due to language limitations.

#### Screening and selection

Two reviewers participated in selection of studies (MT and MP). At the initial phase of selection, abstracts and titles of articles were screened by one reviewer (MT) to exclude articles that clearly were not related to wide-diameter dental implants. The previously described inclusion and exclusion criteria were applied when including articles for full-text screening. When there was a doubt as to the relevance of the article, due to insufficient information in the abstract, the full-text article was analyzed together with a second reviewer (MP).

#### Search results

**Fig. 1:**
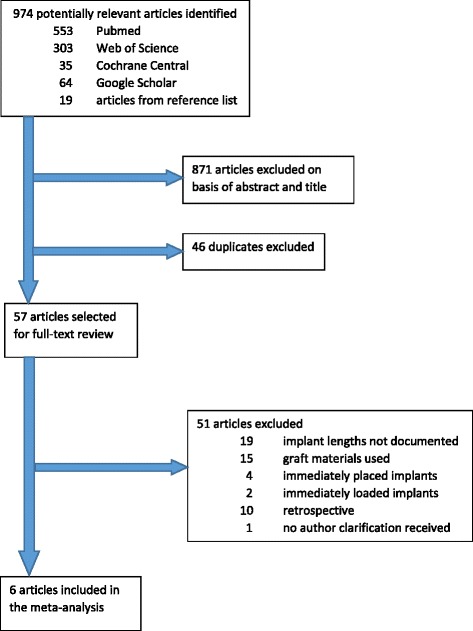
Study selection for wide-diameter implant articles

The search yielded 553 potentially relevant articles in PubMed, 303 in Web of Science, 35 in Cochrane Central Register of Controlled Trials, 64 from Google Scholar, and 19 not identified in the above search strategies from hand searching of reference lists of selected articles (Fig. 1). After screening the abstracts of the articles, 38 articles were selected for full-text screening from PubMed, 26 from Web of Science, 6 from Cochrane Central Register of Controlled Trials, 16 from Google Scholar, and 17 from hand searching of reference list. After elimination of duplicate articles, a total of 57 articles were selected for full-text screening.

Predetermined inclusion and exclusion criteria were applied to the selected full text by two reviewers (MT and MP); article selection was completed independently and then in unison. Areas of ambiguity were resolved through discussion. As for two articles that needed clarification of patient selection criteria, the authors were contacted via email and given 2 weeks to respond. One author responded with the requested information. Six articles remained for further review.

A total of 51 studies were excluded after the full-text review. These included 19 studies that did not document different lengths of wide implants studied, 15 studies that used graft materials in or around the implant sites, 4 studies that immediately placed implants in extraction sites or upon removal of failed implants, 2 studies that immediately loaded the implants, 10 studies that were retrospective, and 1 study that did not receive a clarification from the author.

#### Data extraction

One researcher (MT) extracted the data, and a second researcher (DPD) independently checked the data extraction for accuracy and completeness. The disagreements were resolved by discussion. The data extraction form was pilot tested on a representative sample before applying it to the selected articles.

#### Statistical analysis

The forest plot was used to determine pooled wide-diameter implant survival rate of the selected studies. The funnel plot was used to determine the possibility of publication bias of the selected studies. Heterogeneity of the data was analyzed to determine if the data from the selected studies can be analyzed and if the random effects model can be used in the meta-analysis. In addition, a meta-regression (type III test of fixed effects) was used to evaluate the effects of location, length, and surface on the implant survival.

### Results

Of the six studies selected, three evaluated surface-treated implants and three machined implants (Table 1). The included studies all used similar criteria for implant survival, which was defined as the absence of mobility, pain, and radiolucent lesions. The implant survival was based on the percentage of implants evaluated, and the implant lengths in the studies range from 6 to 16 mm (Tables 2 and 3). The number of patients receiving wide implants was not specified in three studies which also evaluated other diameter implants [13–15]. Only data on the wide-diameter implants in those studies were included in the meta-analysis. Three studies evaluated only wide-diameter implants. The number of patients evaluated in these studies was as follows: Khayat et al. [13] studied 71 patients, Polizzi et al. [16] studied 34 patients, and Schincaglia et al. [17] studied 15 patients. Schincaglia et al.’s study was a randomized controlled trial evaluating immediate-loading versus delay-loading of wide-diameter implants; only data of the control group that was not immediately loaded was included in the meta-analysis. The mean patient follow-up of the six studies ranged from 1 to 8 years. The location of the implants placed in the selected studies (Table 4) was as follows: two studies evaluated implants placed in the posterior mandible [17, 18], one study in the edentulous mandible [14], and three studies in various areas of the maxilla and mandible [13, 15, 16].

**Table 1:** Wide-diameter implants

Implant diameter (mm)	Implant lengths	No. of implants (total)	Implant type	Implant surface	Prospective clinical study	Placement follow-up/mean (range)	Implant survival (%)	Age range (years)
4.7	8, 10, 13, 16	117	Zimmer (Screw vent, Paragon)	Acid-etched, uncoated	Khayat et al. 2001 [13]	Healing 3–6 months plus 17 months loading (11–21 months)	95	–
5.0	7	14	Endopore (Innova Corp)	Sintered porous	Deporter et al. 2001 [18]	32.6 months	100	25–76 (53.7)
5.0	8.5, 10, 11.5	15	Mark III WP (Nobel Biocare)	Ti-unite	Schincaglia et al. 2008 [17]	3–4 months healing plus	100	35–68 (49.2)
						12 months loading		
5.0	6	13	Brånemark (Nobel Biocare)	Machined	Friberg et al. 2000 [14]	8 years (1–14 years)	100	38–93 (63)
5.0	6, 7, 8, 8.5, 10	109	Brånemark (Nobel Biocare)	Machined	Tawil and Younan 2003 [15]	Healing plus 24 months loading	94.5	22–80 (53.6)
5.0	7, 8.5, 10, 11.5	38	Brånemark (Nobel Biocare)	Machined	Polizzi et al. 2000 [16]	36 months	92	29–69

**Table 2:** Wide surface-treated Implants

Study	Implant surface	Implant type	Implant length	No. of implants	No. failed	% survived
Khayat et al. 2001 [13]	Acid-etched, uncoated	Zimmer (Screw vent, Paragon)	8	29	2	93.1
			10	45	4	91.1
			13	28	0	100
			16	15	0	100
Deporter et al. 2001 [18]	Sintered porous	Endopore (Innova Corp)	7	14	0	100
Schincaglia et al. 2008 [17]	Ti-unite	Mark III WP (Nobel Biocare)	8.5	5	0	100
			10	5	0	100
			11.5	5	0	100

**Table 3:** Wide machined implants

Study	Implant surface	Implant type	Implant length	No. of implants	No. failed	% survived
Polizzi et al. 2000 [16]	Machined	Brånemark (Nobel Biocare)	7	2	0	100
			8.5	8	1	87.5
			10	15	1	93.3
			11.5	13	1	92.3
Friberg et al. 2000 [14]	Machined	Brånemark (Nobel Biocare)	6	13	0	100
Tawil and Younan 2003 [15]	Machined	Brånemark (Nobel Biocare)	6	16	0	100
			7	3	0	100
			8	27	1	96.3
			8.5	8	2	75.0
			10	55	3	94.5

**Table 4:** Implants used in the maxilla and mandible

Study	Implant surface	Implant type	No. of implants in maxilla (no. failed)	No. of implants in mandible (no. failed)	% survived in maxilla	% survived in mandible
Khayat et al. 2001 [13]	Acid-etched, uncoated	Zimmer (Screw vent, Paragon)	49 (2)	62 (4)	95.9	93.5
Deporter et al. 2001 [18]	Sintered porous	Endopore (Innova Corp)	0	14 (0)	–	100
Schincaglia et al. 2008 [17]	Ti-Unite	Mark III WP (Nobel Biocare)	0	15 (0)	–	100
Polizzi et al. 2000 [16]	Machined	Brånemark (Nobel Biocare)	4 (0)	34 (3)	100	91.2
Friberg et al. 2000 [14]	Machined	Brånemark (Nobel Biocare)	0	13 (0)	–	100
Tawil and Younan 2003 [15]	Machined	Brånemark (Nobel Biocare)	22 (2)	87 (4)	90.9	95.4

**Fig. 2:**
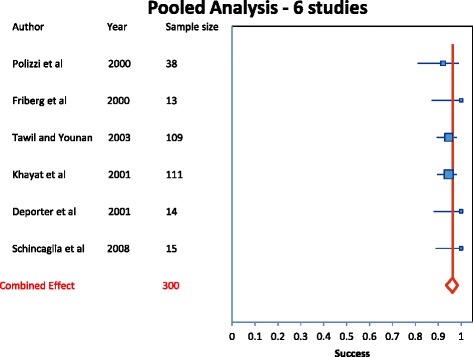
Forest plot

**Fig. 3:**
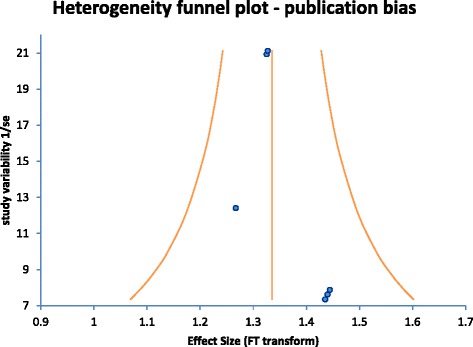
Funnel plot

The forest plot (Fig. 2) showed a pooled wide implant survival rate of 96.3 % (Table 5). The funnel plot (Fig. 3) was analyzed for publication bias. No publication bias was found in the selected studies. The meta-analysis heterogeneity statistics were shown in Table 6. The Q statistic was a measure of the total variance of the studies and along with the p-value showed that the studies do not differ significantly from the mean effect. The I^2^ statistic along with the 95 % uncertainty interval measured the degree of inconsistency among the studies and showed no inconsistencies among the studies. τ​^2^ was a measure of the between study variance and was defined as 0 if the Q value was less than the expected variance (Number of studies -1). The results showed no significant heterogeneity among the included studies. Within the meta-analysis using a random effects model, a meta-regression showed that the fixed effects of location, length and surface did not have a significant effect (*P* > 0.05) on survival (Table 7).

**Table 5:** Meta-analysis implant data—pooled analysis

Authors	Number	Success	ci−	ci+	Weight (%)
Polizzi et al. [16]	38	0.921	0.810	0.990	12.71
Friberg et al. [14]	13	1.000	0.872	1.000	4.46
Tawil and Younan [15]	109	0.945	0.893	0.981	36.14
Khayat et al. [13]	111	0.946	0.895	0.982	36.80
Deporter et al. [18]	14	1.000	0.881	1.000	4.79
Schincaglia et al. [17]	15	1.000	0.888	1.000	5.12
	300	0.963	0.934	0.985	100

**Table 6:** Heterogeneity statistics

*Q*	*P*	
2.7008	0.7460	
*I* ^2^	ci−	ci+
0.00 %	0.00 %	74.62 %
*τ* ^2^	ci−	ci+
0.0000	0.0000	0.0069

**Table 7:** Meta-regression—effect of surface and lengths

Type III tests of fixed effects				
	Num	Den		
Effect	DF	DF	*F* value	*P*
Surface	1	11	0.84	0.3787
Length	7	11	0.97	0.4951
Location	1	11	0.00	0.9868

Degree of free (DF) contributes to the determination of *P* value

### Discussion

The present meta-analysis was limited to prospective clinical studies and utilized a rigorous inclusion and exclusion criteria. Studies included in the analysis were limited to cases in which implants placed in sites with adequate bone volume without grafting. Implants were placed in healed sites and loaded after at least 1–3 months of healing. All studies had at least 1-year follow-up. Patients were required to have adequate health to undergo implant surgery. Controlled medical conditions and smoking status were not excluded. Excluded were studies where implants were placed in sites that were initially deemed to have adequate bone; however, at the time of implant surgery, required the use of bone graft. Data from these studies could not be analyzed due to unclear documentation of which implants were grafted, thus preventing separation of the data for the analysis [19, 20]. The data from some of the survival studies were not able to be analyzed due to the lack of a clear description of implant length. The exclusion of these studies along with the rigorous inclusion criteria limited our meta-analysis to six studies. These six studies were well-documented with clear data on implant length, surface, and location.

The overall survival rate of wide-diameter implants based on the pooled data of the included six studies was 96.3 %, and this was within the reported range of wide-diameter [21] and regular-diameter [22] implant survival rates. Machined implants functionally integrate with the surrounding bone via a macroscopic interlock of the implant threads with the bone. Surface treatment of the machined threads increases the effectiveness of the interlock resulting in an improved bone-to-implant interface [23, 24]. However, our meta-analysis found no significant difference between the implant survivals of machined compared to surface-treated wide-diameter implants. Similarly, Al-Nawas et al. [25] also reported no significant difference between machined and double-etched surface-treated standard-diameter implant survival. Conversely, Maló and Araújo Nobre [26] reported significantly more failures for machined compared to surface-treated narrow (3.3-mm diameter) implants. This suggests that the implant surface characteristics may have an impact on implant survival rate based on the implant diameter, and as the diameter of the implant is increased, as in the wide-diameter implant, this impact may not be statistically significant. It should be noted that stringent inclusion criteria were applied including non-grafted sites, controlled medical conditions, and adequate bone volume.

In the present meta-analysis, the location of wide-diameter implants did not impact survival. This was in agreement with Degidi et al. [27] whose study did not find a statistically significant difference in the survival of wide-diameter implants in varying bone densities in the maxilla and mandible. However, this was contrary to some studies [16, 28] that reported a lower wide-diameter implant survival in the posterior mandible compared to the maxilla. This was postulated to be due to the low marginal bone vascularity of the mandible [29, 30]. And, this was also contrary to some other studies [31, 32], which reported a better outcome for immediately placed implants placed in the mandible because of better bone density and quality.

The various lengths of wide implants used in the six selected study ranged from 6 to 16 mm, and the following implant lengths assessed in the meta-regression were 6, 7, 8, 8.5, 10, 11.5, 13, and 16 mm. Unlike our meta-analysis which focused solely on the wide-diameter implant, very few studies looked at the effects of different lengths on the survival rate specific to the wide-diameter implant. Most studies reported survival rates of the shorter implant lengths (≤10 mm) with varying diameter implants (3.75, 4, 5, and 6 mm) [33]. Studies by Deporter et al. [18, 34] included different diameter implants and were not limited to only wide-diameter implants; these studies also found no significant effect of implant length on implant performance. Conversely, Olate et al. [35], who also included different diameter implants, observed the largest failure in their short implants compared to long or medium implants. However, Olate et al. evaluated 1649 implants retrospectively, 295 were wide-diameter implants (17.9 %), 1217 were regular-diameter (73.8 %), and 137 were narrow-diameter implants (8.3 %). Thus, their conclusion would pertain more to the regular-diameter implants which makes up the majority of implant evaluated in their study. This would seem to indicate that length may have an effect on regular-diameter implant survival, but this would require further investigation. Our meta-regression, which evaluated the prospective data of a total of 306 wide-diameter implants with lengths ranging from 6 to 16 mm, concluded that wide-diameter implants ranging in length from 6 to 16 mm would not have any significant effect on the implant survival. It should be stressed that stringent inclusion criteria were applied for study selection, and hence, these results cannot be generalized to patients with medical or oral compromise.

## Conclusions

This meta-analysis concluded that the location, length, and surface treatment of the wide-diameter implant do not significantly affect its survival. It is therefore suggested with caution that when the conditions of the implant site corresponds to the inclusion criteria used in our meta-analysis, choosing a wide implant in the posterior mandible or maxilla, where implant length may be limited by the nerve or the sinus, the use of a short implant regardless of the implant surface would not adversely affect its survival.
